# Teaching the 6 Criteria of the International Federation of Clinical Neurophysiology for Defining Interictal Epileptiform Discharges on EEG Using a Visual Graphic

**DOI:** 10.1212/NE9.0000000000200073

**Published:** 2023-05-15

**Authors:** Fábio A. Nascimento, Sándor Beniczky

**Affiliations:** From the Department of Neurology (F.A.N.), Washington University School of Medicine, St. Louis, MO; and Department of Clinical Neurophysiology (S.B.), Danish Epilepsy Center, Dianalund and Aarhus University Hospital; Department of Clinical Medicine (S.B.), Aarhus University, Denmark.

The 6 operational criteria for defining interictal epileptiform discharges (IEDs) proposed by the International Federation of Clinical Neurophysiology simplify IED identification into a series of systematic feature extraction tasks. The presence of at least 4 criteria defines a sharp transient as epileptiform.^1^ The criteria displayed in the teaching graphic (Figure) can be used to train novice EEG readers how to critically appraise the components of a sharp transient.^2,3^ The visual-based, systematic teaching method ensures that trainees can accurately and reliably identify IEDs on EEG.^4^ We use this graphic to teach the criteria to trainees and as a visual aid when applying the criteria during supervised EEG reading sessions.

**Figure:**
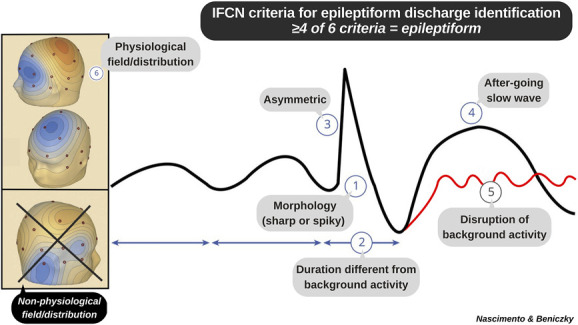
Teaching Image Displaying the 6 Criteria of the IFCN for Identifying Interictal Epileptiform Discharges on EEG (1) Sharp or spiky morphology (20–200 milliseconds); (2) different wave duration than background activity; (3) waveform asymmetry; (4) after-going slow wave; (5) disruption of background activity: flattening or low-voltage alpha or beta frequency activity after (most frequently) or before sharp transient; (6) distribution suggestive of cerebral source/physiologic field. Criteria 4 and 5 are independent. IFCN = International Federation of Clinical Neurophysiology.
